# Understanding Peritoneal Fluid Estrogen and Progesterone Concentrations Permits Individualization of Medical Treatment of Endometriosis-Associated Pain with Lower Doses, Especially in Adolescents Not Requiring Contraception

**DOI:** 10.3390/jcm14207196

**Published:** 2025-10-12

**Authors:** Philippe R. Koninckx, Anastasia Ussia, Leila Adamyan, Arnaud Wattiez, Paola Vigano

**Affiliations:** 1 Departments of Obstetrics and Gynecology, Katholieke University Leuven, 3000 Leuven, Belgium; 2 Departments of Obstetrics and Gynecology, University of Oxford, Oxford OX1 2JD, UK; 3 Departments of Obstetrics and Gynecology, University Cattolica del Sacro Cuore, 00168 Rome, Italy; 4 Gruppo Italo Belga, Villa Del Rosario, 00168 Rome, Italy; anastasia.ussia@gmail.com; 5 Department of Operative Gynecology, Federal State Budget Institution V. I., Kulakov Research Centre for Obstetrics, Gynecology, and Perinatology, Ministry of Health of the Russian Federation, Moscow 117997, Russia; adamyanleila@gmail.com; 6 Department of Reproductive Medicine and Surgery, Moscow State University of Medicine and Dentistry, Moscow 127473, Russia; 7 Latifa Hospital, Dubai P.O. Box 1853, United Arab Emirates; arnaud.wattiez@wanadoo.fr; 8 Department of Obstetrics and Gynaecology, University of Strasbourg, 67000 Strasbourg, France; 9 Gynecology Unit, Fondazione IRCCS Ca’ Granda Ospedale Maggiore Policlinico, 20122 Milano, Italy; paola.vigano@policlinico.mi.it

**Keywords:** endometriosis, hormonal medical therapy, hormone replacement therapy, peritoneal fluid, GnRH, oral contraception

## Abstract

**Objectives**: The aim of this study was to review the importance of peritoneal fluid steroid hormone concentrations to understand the mechanism of hormonal medical treatment of endometriosis-associated pain. **Design**: The study included a PubMed search and a pilot trial in 8 adolescents. **Results**: Oral contraceptives (OCs) were designed to inhibit ovulation in all women, and doses are much higher than the mean ovulation-inhibiting dose. Therefore, in most women, half a dose and in some women, even less is sufficient to inhibit ovulation. The inhibition of ovarian function and ovulation decreases estrogen and progesterone concentrations in plasma and peritoneal fluid. Surprisingly, the effect on peritoneal fluid steroid hormone concentrations has not been considered to explain the impact on endometriosis-associated pain. The lowering of the high estrogen concentrations in peritoneal fluid is sufficient to explain the pain decrease in superficial and ovarian endometriosis. A direct progesterone effect is unlikely, given the high progesterone concentrations in the peritoneal fluid of ovulatory women. In 8 adolescents, half an OC dose resulted in an apparently similar pain relief as a full dose (personal observation). **Conclusions:** The decrease in ovarian and superficial pelvic endometriosis-associated pain with OCs can be explained by lowering the intra-ovarian and the high estrogen concentrations in peritoneal fluid after ovulation. A direct progesterone effect is unlikely. Since OCs are severely overdosed in most women, half a dose is sufficient in most with fewer side effects, permitting individualization of therapy in women not requiring contraception. Understanding peritoneal fluid also explains that hormone replacement therapy is not contraindicated in most women with a history of endometriosis. Since the mechanisms of medical therapy of endometriosis-associated pain and the prevention of progression might be different, the growth of lesions must be monitored during treatment.

## 1. Introduction

Hormonal medical therapy for the treatment of endometriosis-associated pain was initiated in the 1960s. Endometriosis was considered endometrial tissue outside the uterus, and, clinically, endometriosis-associated pain was observed to decrease during pregnancy and during treatment with oral contraceptives (OC), either combined OCs, considered similar to a pseudopregnancy [1,2], or progestogen-only contraceptives [3]. Numerous publications and meta-analyses have described their efficacy in reducing endometriosis-associated pain in 70% of patients [4], and medical therapy is considered the first line of treatment. Medical therapy has been observed to inactivate endometriosis lesions, and is believed to reduce the progression, although the latter is questioned today [5]. The mechanism of action was initially considered a progesterone effect but shifted to a hypoestrogenic effect due to the efficacy of Gn-RH treatments, lowering estrogens without adding progesterone. It remains unclear whether progestins have an additional effect on superficial endometriosis and whether some progestins are more specific in reducing pain.

Our understanding of oral contraception, the menstrual cycle, and endometrial growth began in the early 1970s with the introduction of radioimmunoassays. The human menstrual cycle is known as a precisely timed cascade of events. The decrease in plasma progesterone concentration at the end of the cycle induces an early follicular increase in FSH, stimulating the existing follicle cohort. A race of the fittest results in a primordial follicle with an exponential rise in estrogens, triggering a luteinizing hormone (LH) peak, ovulation and a corpus luteum with progesterone secretion, lasting for 12 days unless rescued by a pregnancy. In women, primates, some mice and bats, an abrupt decrease in plasma estrogens or progesterone concentrations results in withdrawal bleeding [6]. This cascade of events explains that the menstrual cycle typically lasts 28 days, with ovulation occurring approximately 14 days after the decline in progesterone.

The history of oral contraception was characterised by the development of nor-testosterone-derived progestins, with a high solubility and strong ovulation-inhibiting effect, and a search for the minimally effective doses. To ascertain contraception in all women irrespective of BMI and age, the doses used in OCs were initially much higher than three SDs above the mean ovulation-inhibiting dose. OC doses were progressively lowered, although still overdosed in the majority of women. Recently, anovulation was redefined by including women with a luteinized unruptured follicle (LUF) [7]. However, it is not clear whether this will permit reliable contraception and a further reduction in OC doses [8].

The plasma concentrations of estrogen and progesterone during the menstrual cycle, their effects on the endometrium, and the molecular mechanisms involved are well understood [9,10]. Estrogens stimulate growth, and progesterone stops growth and induces secretory changes, preparing the endometrium for an embryo. Less emphasised is the huge individual variability in plasma steroid hormone concentrations, with follicular estrogen concentrations (mean ± 2 SD) ranging from 140 to 400 pg/mL and luteal progesterone concentrations from 8 to more than 20 ng/mL. Indirect evidence suggests that individual women have similar concentrations in successive cycles, indicating an individually variable dose-effect relationship.

The effects of estrogens and progesterone are assumed to be similar in the endometrium and endometriosis, but observed differences challenge this assumption. Endometriosis lesions are clonal [11] as demonstrated for typical [12,13], cystic ovarian [14,15] and deep lesions [16]. The associated pain is highly variable. Individual lesions vary biochemically, with variable aromatase activity and progesterone resistance [17,18]. They exhibit genetic and epigenetic [11,19,20] as well as histological differences, accompanied by variable fibrosis [19,20]. Progesterone resistance must be strong [21] in superficial lesions with a proliferative histology, notwithstanding high progesterone concentrations in peritoneal fluid. Individual glands are microheterogeneous for epithelial cancer driver mutations [22] and estrogen and progesterone receptors [23]. Menstrual bleeding of endometriosis lesions was rarely observed during laparoscopy, and blood remnants, often seen by pathology, are more similar to micro-bleedings in the endometrium during continuous OC or progestin-only therapy. Cyclic changes of steroid hormone receptors in endometriosis are more similar to the basal than the functional layer of the endometrium [24]. The growth of endometriosis lesions is self-limiting, with fibrosis typically serving as the end-stage [25]. A depth deeper than 5 mm under the peritoneum [26] was suggested to define deep endometriosis because of the biphasic frequency distribution of depth with a nadir around 5–6 mm of depth, and since the deeper parts were more frequently in phase with the endometrium [27].

The pathophysiology of endometriosis remains unclear, with hypotheses ranging from the implantation of normal endometrial cells following retrograde menstruation to the metaplastic differentiation of coelomic [28] or stem cells to genetically or epigenetically abnormal cells [29]. If cells are normal, the endocrine and immunologic environment explains the natural history and development, with fibrosis as an end-stage. Hereditary predisposition is considered a risk due to inherited genetic or epigenetic cellular or immunological changes. If the cells are abnormal, the natural history also varies with the acquired epigenetic and genetic abnormalities. The numerous observed differences between endometrial and endometriosis cells suggest that endometriosis cells are (irreversibly) distinct from the endometrium, regardless of the driving mechanism.

**Figure 1:**
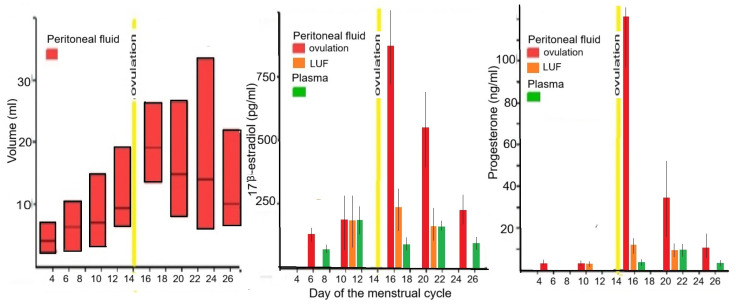
Peritoneal fluid volume and plasma and peritoneal fluid concentrations of estrogens and progesterone during the menstrual cycle in ovulatory women and women with a Luteinized Unruptured follicle (LUF). The peritoneal fluid volume increases during the follicular phase and remains high after ovulation. In the follicular phase, peritoneal fluid 17β-estrogen concentrations are similar to plasma concentrations in ovulatory and LUF women. Progesterone concentrations are almost as high as during the luteal phase in plasma. After ovulation (yellow bar), the steroid hormone concentrations of both 17β-esstradiol and progesterone abruptly increase 5 to 10 times in ovulatory women but not in LUF women. Authors’ creation from [31,36,42,43].

Superficial pelvic endometriosis develops in the non-vascularised peritoneal cavity, [21] which is lined with the peritoneum and in direct contact with the outside world, similar to the mouth. The peritoneal cavity has a specific thermoregulation [30], hormonal microenvironment [31], high iron concentration [32] causing oxidative stress and a different immunology and microbiome [33,34], originating from the outside world through the genital tract and from the intestinal microbiome by diapedesis. The peritoneum regulates the transport of liquids, proteins, leucocytes and even gases such as CO_2_ [35]. The peritoneum is moistened by transudation from plasma, permitting gliding of the bowels, and the volume of peritoneal fluid is minimal in men and women without ovarian activity. In ovulatory women, peritoneal fluid increases by exudation from the growing follicle or corpus luteum [36,37,38,39] up to 200 mL before ovulation (Figure 1). Unsurprisingly, this exudate contains high concentrations of estrogens and progesterone, resulting in follicular estrogen concentrations much higher than in plasma and progesterone concentrations almost as high as in the luteal phase in plasma [31,37,40]. After follicular rupture, the peritoneal fluid concentrations of both estrogens and progesterone increase more than 10-fold [41] in women with and without endometriosis. This abrupt increase in peritoneal fluid estrogen and progesterone concentrations does not occur in women with a LUF, and the concentrations are only slightly higher than in plasma [42,43]. The peritoneum actively regulates the exchange between plasma and the peritoneal cavity, with slower diffusion of larger molecules [43] and a 30% lower concentration of sex hormone-binding globulin and transcortin, resulting in relatively higher free steroid hormone concentrations [31].

The effect of hormonal medical treatment of endometriosis on peritoneal fluid hormone concentrations and on superficial endometriosis lesions has rarely been taken into account. Therefore, we reviewed the effect of hormonal medical therapy for endometriosis on the estrogen and progesterone concentrations in peritoneal fluid, together with an overview of the basics of steroid hormone therapy.

## 2. Materials and Methods

Pubmed was searched for [(“LHRH agonist” OR “LHRH antagonist” OR estroprogestins OR progestins OR “medical therapy”) AND Endometriosis AND mechanism] and for [endometriosis AND (peritoneal fluid OR peritoneal cavity) NOT animal AND (concentration OR estrogen OR progest) AND treatment]. The 204 and 184 articles found were hand-searched for “peritoneal fluid” or “peritoneal cavity”. Peritoneal fluid or peritoneal cavity was mentioned in only 12 articles, but the eventual importance to understand the effect of medical therapy on endometriosis-associated pain was not considered.

In 8 normal weight adolescents, not needing contraception, and taking OCs for suspected (since a normal clinical and transvaginal ultrasound examination) endometriosis-associated pain, the OC dose was halved, and recurrence of pain was observed one month later.

## 3. Results

### 3.1. Basics of Medical Treatment with Estrogens or Progestagens

#### 3.1.1. Tissue Concentrations

**Figure 2:**
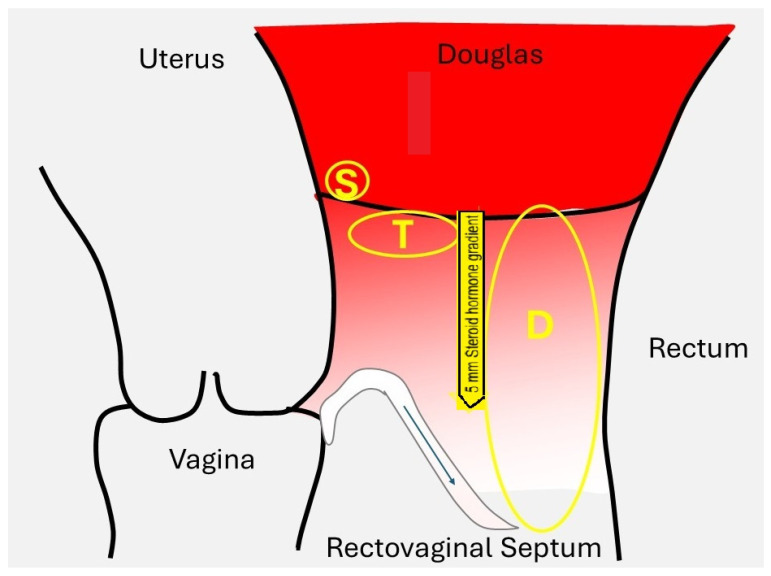
Steroid hormone gradient from peritoneal fluid to plasma. The depth of the gradient has been estimated at approximately 5 to 7 mm, although this varies with the vascularisation of the underlying tissues. The concentrations, illustrated in red, illustrate that subtle (S) and typical (T) lesions and the superficial parts of deep (D) lesions are mainly influenced by peritoneal fluid steroid hormone concentration (authors’ creation), thus explaining the biphasic frequency distribution of depth [29] with the deeper parts more in phase with the endometrium [27].

Steroid hormones are small (around 300 Daltons) lipophilic molecules and, thus, poorly soluble in water.This explains that they must be transported in plasma after binding to specific proteins, such as SHBG, transcortin, and albumin. These protein-bound steroid hormones act as a reservoir, maintaining constant free concentrations at around 1% [44]. Being lipophilic, the free steroid hormones easily cross the lipid bilayer of the cellular membrane [45] and diffuse into the surrounding tissues. The diffusion depth of high peritoneal concentrations must vary with the concentration differences and local blood flow, thereby equilibrating concentrations with those in plasma. The diffusion depth is estimated to range up to 7 mm, although it has not been directly measured (Figure 2).

#### 3.1.2. Dose Effect Relationship

Steroid hormones bind to specific cytoplasmic receptors, which then translocate to the nucleus, where they regulate DNA transcription and protein synthesis. Estrogens induce the progesterone receptor [46] and are a prerequisite for a progesterone effect. The binding of the free hormone (H) to the receptor (R) results in the hormone-receptor complex (HR), which is regulated by the dissociation constant: K_d_ = [H][R]/[HR]. This receptor binding mechanism explains that the maximal effect occurs when the receptor is fully saturated, which translates clinically in the absence of an overdose. Another consequence is that the hormonal effect increases sigmoidally with the logarithm of the concentration [47], that the concentration resulting in a half-maximal effect reflects the potency of the hormone, and that weaker hormones can decrease the effect of stronger hormones by competitive inhibition of binding. Additionally, the dose-effect relationship varies with the complexity of DNA transcription, epigenetic changes, and the specificities of the hormone-receptor complex in different tissues [48]. An example of the different effects of similar hormones in different tissues is the progestin effect on the endometrium, known as the Clauberg effect, and its ovulation-inhibiting effect. The ovulation inhibition effect is relatively stronger for nor-testosterone than for progesterone derivatives, explaining their use in OCs.

#### 3.1.3. Variable Bioavailability

For oral steroid hormone therapy, solubility in water is fundamental. Conjugated steroid hormones dissociate in water and are well-resorbed. The solubility of non-conjugated steroid hormones varies with the number of hydroxyl groups. For example, oestriol, with three hydroxyl groups, can be taken orally; 17β-estradiol, with two hydroxyl groups, and progesterone, without a hydroxyl group, require micronisation to increase the surface area. However, plasma concentrations become less predictable, as they also vary with the degree of micronisation. After oral intake, steroid hormones undergo a first-pass effect in the liver, resulting in locally higher concentrations and subsequent metabolization. For example, estradiol is metabolised into estrone for approximately 98%, which explains the use of ethinylestradiol in oral contraceptives, as the ethinyl group protects the 17β-hydroxyl group from oxidation. Additionally, the role of the intestinal microbiome and the enterohepatic cycle, involving the reabsorption of hydrolysed conjugated steroid hormones secreted into the bile, is variable. The intake of estrogens and progesterone also affects the intestinal microbiome, but the clinical consequences are not yet clear [49].

#### 3.1.4. Oral Contraceptives Are Overdosed in Most Women

**Figure 3:**
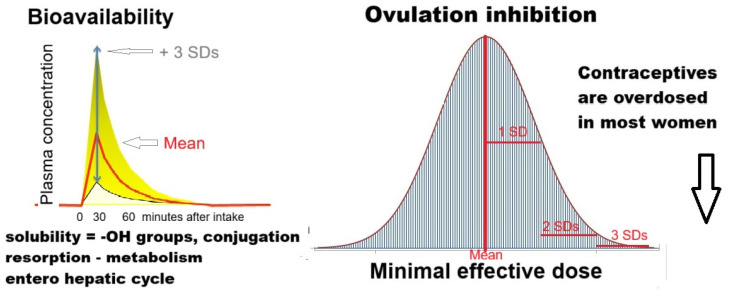
Estrogens and progestins in oral contraception. The bioavailability of ethinylestradiol varies at least fourfold [49,50,51,52,53], due to variations in solubility, resorption, metabolism, the enterohepatic cycle, and possibly the microbiome. The minimal effective dose to inhibit ovulation varies with the bioavailability and individual characteristics. To be contraceptive in all women without failures, the doses used are much higher than 3 standard deviations above the mean minimal effective dose. This explains the progressive decrease in OC doses from 50 µg EE to 30 and 20 µg EE over time.

The effect of administered hormones is usually described as the average effect or the average plasma concentrations in the average woman (Figure 3). Unfortunately, average effects do not reflect the variability in individual women’s resorption, metabolism, and plasma concentrations. This variability is reflected in plasma concentrations that vary at least 4-fold after oral intake.

An example is the fourfold increase in variable plasma concentrations after the oral intake of ethinylestradiol (EE) [50,51,52,53,54]. Considering the difficulty of the EE assay and the logarithmic distribution of plasma hormone concentrations, it is estimated that the oral intake of 50 µg ethinylestradiol results in plasma concentrations ranging from 100 pg/mL, which is the lower limit required for contraceptive efficacy, to 1000 pg/mL, causing morning nausea. An example of individually variable biological effects is the relationship between plasma estrogen concentrations and menopausal symptoms. Below 50 pg/mL of estradiol, most women have menopausal symptoms, but more than 180 pg/mL is needed before all women are symptom-free. The variable bioavailability necessitates individualised therapy, which, unfortunately, is unrealistic for the pharmaceutical industry. OCs are designed to be effective in all women, irrespective of their weight, resorption or metabolism. Therefore, the doses used in OCs are higher than three standard deviations above the mean minimal effective dose and, therefore, overdosed in the large majority of women.

Since it is probably not relevant for hormonal therapy of endometriosis, we will not discuss the brain effects of metabolites, such as 5-α-reduced pregnanes [55], nuclear retention times, plasma versus biological half-lives, and fast resorbable conjugated steroid hormones with a peak concentration after 30 min versus micronised steroid hormones with a slow resorption and peak concentrations after 5 h.

#### 3.1.5. Conclusions for Steroid Hormone Basics

The bioavailability of estrogens and progestogens after oral intake is highly variable in individual women, due to factors such as solubility, resorption, liver metabolism, and possibly the microbiome.

Estrogens and progestins do not have an overdose: the effect increases sigmoidally with the logarithm of the concentration up to saturation of all receptors.

To obtain a reliable contraception for all women, oral contraceptives are severely overdosed in most women.

### 3.2. Estrogen and Progesterone Concentrations in Endometriosis Lesions

The effect of estrogens and progestogens on endometriosis varies with local concentrations that reflect plasma or peritoneal fluid concentrations, aromatase activity, estrogen and progesterone receptors [55], and progesterone resistance [17]. The concentrations of estrogens and progesterone in plasma and the much higher concentrations in peritoneal fluid (Figure 1) are well documented (Figure 2). In addition, the local peritoneal fluid concentrations are even higher around the ovary with the growing follicle, which is important for oocyte pick-up [56], and in the right diaphragm because of peritoneal fluid circulation, probably explaining the higher incidence of endometriosis in comparison with the left This is a conclusion of steroid hormone basicdiaphragm. The intra-ovarian [57] and oviductal concentrations are 5 to 10 times higher [58] than plasma concentrations because of local secretion and the countercurrent exchange between the ovarian artery and vein [59,60,61].

Some observations in endometriosis are easier to explain if local concentrations are considered. Strong progesterone resistance must be postulated for the many superficial endometriosis lesions, which are proliferative, despite progesterone concentrations in peritoneal fluid exceeding 100 ng/mL [21]. Concentrations in cystic ovarian endometriosis explain that the endometriosis cells are generally inactive but can become proliferative after opening and draining large endometriosis cysts during two-step surgery (unpublished observations, PK). The high concentrations in the ovarian artery contribute to the ovarian concentrations and probably explain some (cystic) oviductal lesions and uterine adenomyosis. Peritoneal fluid concentrations explain that superficial parts of deep endometriosis are more frequently inactive, while the deeper parts are more proliferative and in phase with the endometrium [27,62]. Aromatase activity in endometriosis lesions could explain the development of deep endometriosis lesions after menopause, even without the administration of exogenous estrogens [63].

### 3.3. Hormonal Medical Therapy for Endometriosis

The clinical efficacy of pregnancy or during therapy with OCs, progestogen-only, or more androgenic progestins such as Danazol and Gestrinone [64] on pelvic endometriosis-associated pain was historically understood as a progesterone effect. These therapies inactivate and shrink lesions, but their activity rapidly resumes after discontinuation of the drug [65]. The efficacy of GnRH agonists and antagonists, which strongly decrease estrogen and progesterone secretion without adding progestins, suggests a hypoestrogenic effect. Without addressing minor differences between drugs containing different progestins, the overall efficacy on pelvic pain is around 70%, even in women with deep endometriosis [5]; however, 10% to 30% of women experience little or no reduction in pain [4].

All medical hormonal therapies of endometriosis, OCs, progestogen monotherapy [66,67,68], and Gn-RH [69] agonists [70] or antagonists [71] inhibit ovulation and decrease ovarian steroid hormone secretion. The plasma concentrations of the administered ethinylestradiol and progestins are poorly documented. The specific assays are rarely available outside the pharmaceutical industry, and the reported series are small [72,73,74]. The concentrations in peritoneal fluid are likely similar to those in plasma, but have not been measured directly, as the volume is too low in women without ovarian activity. All data confirm the expected wide variability of peak concentrations a few hours after intake. Peak estrogen concentrations appear to be physiological between 100 and 300 pg/mL. It is challenging to estimate the direct effect of the administered progestins on endometriosis, as nor-testosterone derivatives are less potent in this regard. It is unclear whether inducing a LUF syndrome without follicular rupture [7] and with much lower estrogen and progesterone concentrations in peritoneal fluid [43] affects superficial endometriosis-associated pain. It is unlikely that a LUF will affect pain caused by cystic ovarian endometriosis.

GnRH antagonists or agonists strongly decrease endometriosis-associated pain, suggesting that the effect on pain results from the lower estrogen concentrations in plasma or peritoneal fluid. Aromatase inhibitors can inhibit estrogen production [75,76], but it is unknown whether their efficacy results from reduced ovarian secretion or synthesis in endometriosis lesions. They must be combined with ovulation inhibitors since, when given alone, the decreased estrogen secretion stimulates gonadotropin secretion [77].

In conclusion, the efficacy of OCs, progestagen monotherapy, or GnRH agonists or antagonists on endometriosis-associated pain appears to result from low estrogen concentrations in the ovary, plasma and peritoneal fluid, rather than from a direct progestagenic effect. The similar efficacy of GnRH agonists with and without add-back therapy [78,79] suggests that for superficial endometriosis lesions, the pain reduction results mainly from reducing the high estrogen concentrations in peritoneal fluid, and that the threshold of estrogen concentrations inducing pain is higher than physiological concentrations. We can only speculate about the Reduction In Pain in endometriosis lesions influenced by plasma concentrations, rather than peritoneal fluid concentrations. A direct progestin effect cannot be excluded, and the threshold of estrogen concentrations causing pain might be different, which is consistent with endometriosis lesions being clonal and heterogeneous.

### 3.4. Individualization of Therapy

Since contraceptives are overdosed in the large majority of women (Figure 3), a 2 to 4 times lower dose should be sufficient for anovulation in most, especially in those with a low BMI. If the pain reduction from superficial endometriosis results from the huge oestradiol concentrations of more than 1000 pg/mL in peritoneal fluid after follicular rupture, inducing a LUF syndrome might be sufficient to decrease pain [80].

Pain relief with half a dose of OC was apparently similar in the 8 adolescents taking OCs for suspected endometriosis-associated pain (observation PK).

### 3.5. Hormone Replacement Therapy After Menopause and Endometriosis

It is unclear whether a history of endometriosis is a contraindication for hormone replacement therapy (HRT) [81]. Besides limited anecdotal clinical observations, there is little evidence that HRT stimulates the growth of endometriosis lesions [82]. Although guidelines do not recommend HRT, two-thirds of practitioners continue to prescribe HRT, apparently without endometriosis being stimulated [83]. These observations are consistent with the Relugolix Spirit studies, in which adding 1 mg estradiol and 0.5 mg norethisterone did not affect the results of pelvic pain reduction [78,79].

## 4. Discussion

Steroid hormone concentrations in peritoneal fluid are important to understand the hormonal therapy of endometriosis. Unfortunately, the physiology of the peritoneal cavity has been poorly studied. The vast literature, comprising over 25,000 publications, primarily describes pathologies such as ascites, dialysis, cancer, peritonitis, adhesion formation, and surgery. The volume of peritoneal fluid [36], the steroid hormone concentrations [31], the LUF syndrome and its association with endometriosis were hot topics in the early 1980s. However, the interest decreased without direct clinical applications and since the hormonal assays of peritoneal fluid require specific laboratory skills. The radioimmunoassays of steroid hormones require extraction to avoid protein and matrix effects, and thin-layer chromatography to prevent interference from related hormones, such as 20α-hydroxyprogesterone, 17α-hydroxyprogesterone, and a series of other unidentified Δ4-3-keto steroids, which are present in high amounts [31]. Only recently [84], using gas chromatography and mass spectrometry, the high progesterone concentrations were confirmed, and over 20 different 20α- and 11α-hydroxylated and 5α-reduced metabolites were described. Unfortunately, it remains unclear whether these metabolites are biologically active and whether they influence superficial endometriosis-associated pain.

Hormonal therapy decreases endometriosis-associated pain by low estrogen concentrations without necessarily a specific progesterone effect, given the efficacy of GNRH therapy. A similar efficacy with and without add-back therapy [78,79] suggests that the primary effect is the reduction in the high peritoneal fluid estrogen concentrations, which exceed 1000 pg/mL after ovulation. Giv84en the very high progesterone concentrations in peritoneal fluid in ovulatory cycles [21], an additional or specific effect of the administered progestins is unlikely [5] in superficial endometriosis. However, other mechanisms that decrease pelvic pain cannot be excluded, such as an effect on the endometriosis-associated low-grade inflammation, characterised by more activated macrophages and their secretory products in the peritoneal fluid. This might contribute to the inhibition of blood vessel growth and an anti-inflammatory effect. It is unclear whether the in vitro effects of dienogest or medroxyprogesterone acetate on aromatase expression and 17β-hydroxysteroid dehydrogenase [10] are clinically relevant.

Since oral contraceptives are overdosed, anovulation can be achieved in most women with much lower doses and thus with fewer side effects. This is important for all women who do not need contraception, especially in adolescents with a low BMI. It is beyond the scope of this manuscript to discuss the contraceptive reliability of individualising the dose and of monitoring ovulation, e.g., with ultrasound exams. The preliminary observations will have to be confirmed in trials, but unfortunately, individualisation of therapy will be difficult since trials traditionally estimate the average effect in the average woman.

The mechanisms that inhibit endometriosis progression probably differ from those that inhibit pain. Endometriosis can develop after menopause in women not taking estrogens [63] with initially little pain. Very severe endometriosis is often found during surgery in women who have been taking OCs for many years. Therefore, yearly follow-ups are indicated during treatment to detect any eventual growth of endometriosis by expert ultrasonographers.

With 70% responders and 30% poor responders, medical therapy is a first-line treatment in women with pelvic pain and suspicion of endometriosis. It is unclear why pain reduction is limited or absent in up to 30% of women. Central sensitisation [85,86], the heterogeneous distribution of estrogen and progesterone receptors [25], and a different dose-effect relationship, i.e., an estrogen-pain relationship, may play a role. Finally, it seems wise to differentiate the effect of hormonal medical therapy on endometriosis-associated pain and the inhibition of endometriosis growth, probably involving different mechanisms. Therefore, the growth of endometriosis during hormonal medical therapy should be closely monitored. If pain relief is insufficient after three months, the diagnosis should be reconsidered because of the many other causes of pelvic pain, such as venous congestion, adenomyosis and vascular pain, e.g., the Nutcracker syndrome [87,88,89]. Surgery is also an option [90].

Robust data demonstrating a contraindication to HRT in women with a history of endometriosis are not available [81]. I (PK) never restricted HRT in women with a history of endometriosis and rarely observed an effect on pelvic pain. However, out of prudence, continuous combined therapy was given, also in women without a uterus. This might have been unnecessary.

## 5. Conclusions

The high concentrations of estrogens and progesterone in peritoneal fluid, especially after ovulation, are fundamental to understand the medical treatment of endometriosis-associated pain. The efficacy of hormonal therapies for superficial pelvic endometriosis results mainly from decreased estrogen concentrations in the peritoneal cavity, with little direct progesterone effect. This concept might differ for the deeper parts of deep endometriosis and other endometriosis lesions, which are mainly influenced by plasma concentrations. Clinically important is that anovulation can be achieved with much lower doses of OCs in most women, especially in adolescents with lower BMI. This concept of peritoneal fluid also explains that a history of endometriosis is not a contraindication for HRT. Since endometriosis may progress during medical therapy, it is essential to distinguish between pain and endometriosis growth and evaluate lesion growth during treatment.

## Data Availability

No new data were created or analyzed in this study.
